# Emotionally adaptive support: a narrative review of affective computing for mental health

**DOI:** 10.3389/fdgth.2025.1657031

**Published:** 2025-10-15

**Authors:** Michelle Schlicher, Yupei Li, Sunil Munthumoduku Krishna Murthy, Qiyang Sun, Björn W. Schuller

**Affiliations:** ^1^ CHI – Chair of Health Informatics, TUM University Hospital, Munich, Germany; ^2^ MCML – Munich Center for Machine Learning, Munich, Germany; ^3^ GLAM – Group on Language, Audio, & Music, Imperial College London, London, United Kingdom; ^4^ MDSI – Munich Data Science Institute, Munich, Germany

**Keywords:** affective computing, digital mental health interventions, artificial intelligence, affective adaptation, emotion recognition

## Abstract

Digital mental health interventions (DMHIs) have become increasingly prominent as scalable solutions to address global mental health needs. However, many existing tools lack the emotional sensitivity required to foster meaningful engagement and therapeutic effectiveness. Affective computing, a field focused on designing systems capable of detecting and responding to human emotions, offers promising advancements to the emotional responsiveness of these digital interventions. This narrative review examines how affective computing methods such as emotion recognition, sentiment analysis, emotion synthesis, and audiovisual and physiological signal processing, are being integrated into DMHIs to enhance user engagement and improve clinical outcomes. The findings suggest that emotionally adaptive systems can strengthen user engagement, simulate empathy, and support more personalized care. Early studies indicate potential benefits in terms of symptom reduction and user satisfaction, though clinical validation remains limited. Challenges such as algorithmic bias, privacy concerns, and the need for ethical design frameworks continue to shape the development of this emerging field. By synthesizing current trends, technological advancements, and ethical considerations, this review highlights the potential of affective computing in digital mental health and identifies key directions for future research and implementation.

## 1 Introduction

Affective computing, which is considered as the development of computational systems that detect, interpret, and respond to human emotions (1), has rapidly advanced digital mental health (2–4). Emotion-aware technologies are increasingly used to personalize care, enhance engagement, and improve outcomes across psychological conditions. Digital mental health interventions (DMHIs), which encompass technology-based tools such as mobile apps, wearable sensors, and conversational agents, aim to assess, monitor, or treat mental health disorders (5, 6). When integrated with affective computing, these systems gain the ability to dynamically adjust to users’ emotional states, offering more responsive and individualized support (7). From stress-sensing wearables (8) to sentiment-aware chatbots (9), this fusion reshapes human–technology interaction, particularly in clinical contexts where emotional understanding is critical to therapeutic success.

Despite its growing importance, comprehensive reviews focused specifically on affective computing within digital mental health remain scarce. Existing literature tends to either survey affective computing broadly without focusing on psychological interventions (10–12), or examine DMHIs without considering the application or the underlying mechanisms of affective computing (13–15). As a result, the unique opportunities, challenges, and ethical implications of integrating affective computing into mental health care remain underexplored.

This narrative review addresses that gap by critically examining emotionally responsive DMHIs—systems that extend beyond emotion detection to adapt their behavior in real time based on users’ affective states. We present several use-cases targeted directly at patients, but also at supporting clinicians. The focus is on monitoring systems and AI-driven chatbots, while evaluating their technical foundations, clinical evaluation, and level of emotional reactivity.

By centering on emotionally adaptive systems, we aim to bridge the gap between computational emotion modeling and the practical realities of digital mental health care. We also highlight ethical considerations, including transparency, emotional manipulation, privacy, and cultural validity. Ultimately, this review offers an interdisciplinary roadmap for researchers and clinicians seeking to responsibly apply affective computing to improve digital mental health outcomes.

## 2 Methods

To investigate annual publication trends and describe the growth of research at the intersection of affective computing and mental health, a structured literature search was conducted using the Web of Science Core Collection and Scopus databases; with 2,106 and 3,353 results respectively. Query terms were applied to titles, abstracts, and keywords. The search period spans from 1997, when Rosalind Picard first introduced the term affective computing (1), to the end of 2024. To focus on computational models designed to train machines in emotion recognition, rather than on studies aimed at understanding human emotion recognition abilities, we restricted the subject areas to Computer Science and Engineering. Duplicate records (2,569) were identified and removed using the systematic review tool Rayyan (16). The search strategy is summarized in Table 1.

**Table 1:** Search strategy for bibliometric analysis of publications at the intersection of affective computing and mental health.

Category	Search terms/filters
Search keywords	
Affective computing	Affective recognition OR mood recognition OR affective computing OR artificial emotional intelligence OR emotion AI OR expression recognition OR emotion recognition OR emotion learning OR sentiment analysis OR sentiment recognition
Mental health	Mental health OR psychological wellbeing OR mental wellbeing OR emotional wellbeing OR mental state OR psychological health OR depression OR anxiety OR stress OR mood disorder OR bipolar disorder OR psychological stress
Web of science (WoS) filters	
Document types	Article, proceedings paper, review article, early access
Categories	Engineering, computer science, informatics, robotics
Scopus filters	
Document types	Article, conference paper, conference review, review
Subject area	Computer science, engineering

## 3 Background

To highlight the growing applicability and scientific interest in affective computing within digital mental health, a comprehensive overview of its evolution is provided, followed by an examination of its key components: emotion modeling, sensing, and adaptation. This foundation offers the necessary context for understanding current challenges and emerging trends in affective computing-based technologies for DMHIs.

### 3.1 The evolution of affective computing in digital mental health

**Figure 1:**
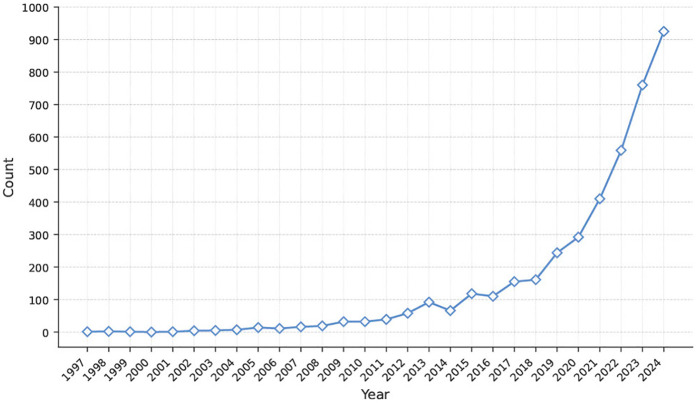
Annual publication counts from Web of Science and Scopus illustrating the growth of research at the intersection of affective computing and mental health from 1997 to 2024.

To provide an illustrative overview of the rapidly increasing trend in annual scientific production concerning affective computing and mental health, the progression of publications is shown in Figure 1. Prior to 2010, the number of publications was sparse, reflecting the early theoretical phase of affective computing, which had yet to find concrete applications in mental health. This first stage (1997–2010) was summarized by Picard’s seminal work, which formally defined affective computing as the study of systems capable of recognizing, interpreting, expressing, and regulating human emotions (1). During this phase, research focused primarily on human–computer interaction, with Ekman’s theory of six basic emotions (17) serving as the primary labeling framework. Early studies explored various overt modalities: Lyons et al. (18) applied Gabor wavelets to facial image analysis; Schuller et al. (19) employed prosodic and acoustic features to classify speech-based emotion; and Pang et al. (20) introduced machine learning for sentiment polarity detection in textual film reviews to give but a few examples. The Kismet robot developed at the MIT Media Lab (21) integrated facial motor expressions and vocal prosody, demonstrating a prototype of “affective dialogue” between humans and machines. While foundational for validating whether machines could perceive emotional cues, these efforts remained largely confined to controlled environments and healthy populations, with limited integration into real-world mental health contexts or deeper affective state modeling.

The second stage (2011–2017) marks a shift from theoretical exploration to practical application. In 2010, the launch of the IEEE Transactions on Affective Computing signified the formal recognition of affective computing as an independent research domain comprising multiple subfields (22). This milestone elevated the academic standing of the field and laid the groundwork for its expansion into healthcare and mental well-being. This is reflected in a steady increase in publications over these years. On the technical front, the proliferation of mobile devices and wearable sensors provided a viable data foundation for affective computing in digital mental health. For example, devices like the Empatica E4 enabled continuous capture of physiological signals such as electrodermal activity and photoplethysmography (23). Similarly, the Affectiva SDK facilitated real-time facial expression analysis on mobile platforms, expanding the capacity for in-situation emotion sensing (24). These technologies led to the development of several representative datasets, including DAIC-WOZ for assessing anxiety, depression, and PTSD symptoms (25); AVEC 2016 for multimodal depression risk modeling (26); and SWELL-KW for stress recognition and contextual behavior analysis (27). Based on these datasets, a number of empirical studies related to mental health have been conducted (28–30).

This period also benefited from increased accessibility to traditional machine learning techniques. Models based on support vector machines, random forests, and k-nearest neighbors were widely used for early affect classification across speech, text, and physiological modalities (31). However, these approaches typically relied on static feature representations and lacked the capacity to model temporal dynamics, contextual dependencies, or individual variability in affective responses. As noted by D’Mello (32), affective systems that do not account for emotion trajectories and situational antecedents fall short of the needs within mental health contexts. This limitation underscored a broader debate within the field: whether affective computing should remain focused on perceptual signal classification or evolve toward deeper emotional intelligence, involving causal modeling and semantic understanding. Since 2018, affective computing has entered a new phase in digital mental health, driven by the adoption of deep learning. As these methods matured, various neural architectures, including convolutional neural networks, recurrent neural networks, and transformers, have increasingly been applied to recognizing emotion-related mental health conditions such as depression, anxiety, and stress, as well as to clinical alignment tasks. Rejaibi et al. (33) employed MFCC features and a recurrent neural network on the DAIC-WOZ depression detection task. Their model achieved state-of-the-art performance at the time, demonstrating the practical value of acoustic features for predicting emotional severity. Ray et al. (34) proposed a multi-layer attention network for AVEC 2019, using convolutional neural networks to extract audio features and LSTMs for textual encoding. A multi-level attention mechanism fused audio, visual, and textual inputs, achieving notable improvements over the original baselines in depression regression. Wu et al. (35) developed a transformer-based self-supervised learning framework for emotion recognition using wearable signals. Their results demonstrated superior performance in classifying stress-related states compared to fully supervised methods, with improved generalizability.

More recently, transformer and then large language models (LLMs) have begun to play a role in affective computing for digital mental health (36). In social media contexts, Bucur et al. (37) introduced a time-enhanced multimodal transformer model that integrates CLIP image embeddings with EmoBERTa textual representations and applies time2vec encoding to model posting intervals, aiming to predict user-level depression risk. The model achieved state-of-the-art F1 scores of 0.931 on a popular multimodal Twitter corpus and 0.902 on the MultiRedditDep dataset. Additionally, LLMs have been used for interactive emotional intervention (3). These technological advancements, along with increased recognition of mental health due to the COVID-19 pandemic, explain the exponential growth in scientific output after 2018.

In sum, the evolution of affective computing in digital mental health reflects not only algorithmic progress but also the beginning of a broader shift from recognizing surface-level emotional cues to aspiring to understand their generative mechanisms and enabling adaptive interventions. While much of the field still relies on direct mappings between (multimodal) cues and emotion states, the increasing integration of context, temporality, and personalized dynamics suggests a transition toward richer, semantically informed affective computing; moving from the perceptual layer (“What you feel”), through the modeling layer (“Why you feel”), toward the intervention layer (“What should be done”). Therefore, affective computing now plays an increasingly central role in the development of digital mental health closed-loop systems, which are cybernetic frameworks that continuously sense mood, update personalized models, and deliver adaptive interventions in real time (38, 39). Nonetheless, realizing this loop still presents substantial theoretical and methodological challenges.

### 3.2 Technical key components of affective computing

We complement existing works, such as the systematic review by Wang et al. (12) and the survey by Afzal et al. (40), which comprehensively cover the technical properties and quantitative performance of key affective computing methods across various modalities like voice and text, by introducing affective computing from a human–computer interaction perspective.

#### 3.2.1 Emotion modeling

Emotion modeling provides the theoretical foundation for affective computing systems to understand human psychological states. Current approaches are primarily grounded in two major emotion theories. Basic emotion theory categorizes emotions into discrete classes, such as Ekman’s six universal emotions (17). Due to its clear label structure and low annotation cost, this framework has been widely adopted in classification tasks across speech, image, and text modalities. It is also integrated into many mental health monitoring systems. However, the theory originates from Western emotional expressions and fails to capture the diversity of emotional signals in clinical or cross-group contexts: Pampouchidou et al. (41) found notable performance differences across gender subgroups in automatic depression detection using discrete labels; Alghowinem et al. (42) further showed that models trained on single-cultural datasets underperform in cross-cultural settings. This can be explained by the currently dominating opinion that emotions are not entirely universal across cultures, but can be perceived differently (43). However, basic emotion theory does not account for such variability. Additionally, there is strong evidence that Ekman’s six emotions do not sufficiently capture the complexity of facial expression (44–46), highlighting the limitations of this framework.

Alternatively, the dimensional emotion theory following Wilhelm Wundt’s foundational work represents emotional states along three axes (valence and arousal, which are popularly used) and sometimes a third axis of dominance (47). This framework is widely used in subfields of affective computing such as speech emotion recognition (48) and text-based emotion recognition (49), where it serves as a regression target in deep learning models. In digital mental health, several studies have extended this approach to clinical emotion modeling. For instance, Ahmed et al. (50) used wearable physiological data (e.g., electrodermal activity, heart rate variability) to predict both depression severity and valence—arousal scores. Their tri-modal model reached high accuracy and F1 score performance for severity classification and valence detection, demonstrating the feasibility of dimensional emotion tracking, though explainability and annotation costs remain problematic.

Beyond these two mainstream models, compound emotion theory was proposed by Du et al. (51). It argues that humans express not only basic emotions but also blends of them, such as “joy–surprise” or “anger–surprise.” Their team identified 21 compound emotions using the Facial Action Coding System (FACS), showing distinct muscular patterns and high inter-rater agreement. While empirical applications in mental health remain limited, compound emotions offer a more fine-grained representational framework for clinical emotion monitoring. Nevertheless, a certain facial movement can express more than one emotional category and conveys besides emotional further social information (52). Similarly, the psychological constructionist theory proposes that emotions are made of several components that are not specific to emotion such as sensory stimulation (53). These different perspectives show that modeling emotions is far from trivial and can contain additional layers that are currently rarely considered in affective computing. Therefore, emotion modeling in digital mental health must carefully consider which psychological framework is most appropriate in the given setting and try to balance label interpretability, expressive granularity, and cross-cultural adaptability.

In current digital mental health applications, emotion modeling typically serves as a theoretical and representational backbone rather than a direct supervision signal. Most clinical systems rely on standardized psychological scales as primary labels or evaluation endpoints, such as PHQ-9 for depression, GAD-7 for anxiety, and DSM-5 for PTSD (54). These instruments quantify persistent symptom severity, whereas emotion models capture momentary affective dynamics. Some studies have begun to explore how emotional labels can complement or predict changes in longer-term symptom scores (55, 56). This dual-layer structure, combining real-time emotion inference with validated clinical assessment tools, offers a promising direction for personalized, longitudinal mental health monitoring.

#### 3.2.2 Emotion sensing and recognition

Emotion sensing and recognition constitutes a central component of affective computing based on emotion modeling. It aims to objectively identify and quantify individuals’ emotional states through multimodal signals. Facial expression analysis today typically employs deep neural network architectures, increasingly enhanced by self-supervised pretraining methods to extract salient static and dynamic features (57). Speech emotion recognition has evolved from traditional acoustic representations (e.g., MFCCs, mel-spectrograms) combined with deep models to capture local temporal patterns, to the use of pretrained frameworks such as wav2vec 2.0 for richer contextual modeling (58, 59).

Physiological emotion modeling leverages biosignals such as electrodermal activity and photoplethysmography, with deep learning techniques used to construct personalized affective representations (60). Text-based emotion recognition has also advanced significantly. Early approaches relied on handcrafted features such as bag-of-words, TF-IDF, and sentiment lexicons. More recent models based on pretrained language transformers (e.g., BERT, LLaMA) have achieved superior performance by capturing semantic ambiguity and emotional metaphor through contextual learning and prompt-based adaptation (61).

Additionally, multimodal systems integrate visual, vocal, physiological, and textual cues, often employing cross-attention or alignment mechanisms to achieve semantic fusion (62). Empirical studies suggest that multimodal approaches generally outperform unimodal models, showing promise in modeling complex psychological states and facilitating more precise affective alignment (63).

Despite these advances, the generalizability of emotion sensing techniques to clinical settings remains limited. For example, a cross-corpus study found that emotion recognition models trained exclusively on data from healthy individuals performed at or below chance level when applied to clinical corpora such as DAIC-WOZ. Minimal fine-tuning on target data was necessary to restore performance, underscoring the presence of strong distributional shifts between source and target domains (42).

Further studies reveal that environmental variables, such as background noise and room reverberation, can reduce unweighted recall by up to 20%, indicating limited robustness in real-world deployment (64). These results suggest that general-purpose models cannot be reliably applied to populations with conditions such as anxiety, depression, or PTSD, where emotional expression, linguistic style, and physiological responses often diverge markedly from those of healthy individuals. Moreover, pharmacological interventions may further alter these manifestations (65), potentially rendering standard annotation schemes inadequate or biased. To address these challenges, it is necessary to adopt strategies such as few-shot adaptation, domain transfer, and subject-specific baseline modeling, at both the representation and label-design levels, to improve robustness, fairness, and interpretability in clinical applications.

#### 3.2.3 Affective adaptation

Affective adaptation, also referred to as emotional responsiveness, describes the ability of affective computing systems to dynamically adjust their behavior or responses in real time based on a user’s emotional state and context. Unlike passive emotion recognition, affective adaptation enables personalized and contextually relevant interactions. This is typically achieved through techniques such as domain adaptation, few-shot and/or fine-tuning, or individual baseline calibration (66, 67).

Emotionally adaptive virtual agents such as Woebot dynamically adjust their dialogue strategies to deliver empathetic and psychologically safe interactions (68). These systems demonstrate that affective adaptation enhances not only model performance but also user engagement, trust, and therapeutic effectiveness. As such, affective adaptation represents a critical step in moving from emotion sensing to active, personalized intervention in digital mental health.

However, cross-domain studies have shown that facial and textual emotion recognition models experience substantial performance degradation when cultural or population-specific variations are not accounted for. Domain adaptation methods have proven effective in mitigating these biases and restoring model performance (69). In physiological modalities, multi-source alignment frameworks (particularly for EEG and electrodermal activity) have enabled successful cross-subject transfer while reducing reliance on large-scale annotations (70). These findings highlight that general-purpose models alone are insufficient to capture the emotional variability observed in clinical populations. As such, adaptation techniques are essential for improving robustness, fairness, and inclusivity in emotion-aware digital health systems.

## 4 Affective computing applications in the mental health domain

Building on the historical and technical background, we now turn to a structured overview of how affective computing is applied across different use cases in digital mental health.

### 4.1 Typology

**Figure 2:**
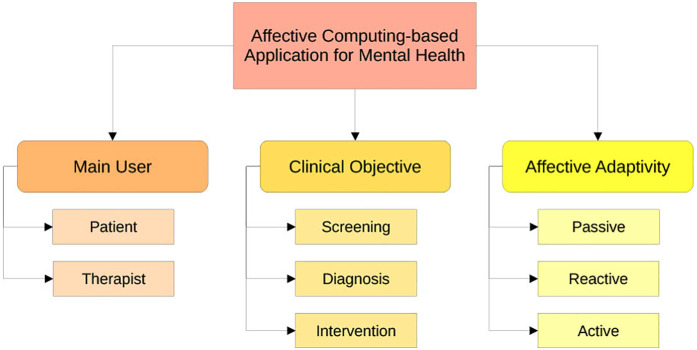
Topology for affective computing-based systems in mental health care.

To structure the possible applications of affective computing in digital mental health systems, we define a typology considering the following dimensions: for whom the system is primarily designed, what the purpose of the system is, and how it responds to the patient’s emotional states. An illustration of this typology can be seen in Figure 2, where the first dimension distinguishes between patient-facing systems, which are used directly by individuals for self-monitoring or therapeutic support, and therapist-facing systems, which assist clinicians with affective insights or decision support.

The second dimension differentiates between the clinical objectives: early identification via screening, detection of existing mental disorders, and interventions to improve the mental state. Often, several of these goals are coupled in one system, as for instance, screening the current state of the patient enables adaptive interventions. Affective computing can be leveraged in screening by automatically detecting subtle emotional and behavioral markers indicative of early mental health changes through multimodal sensing. For diagnosis, it can provide objective, continuous assessments of emotional states to complement clinical evaluation, helping to identify symptom severity or subtype differentiation. In intervention, affective computing enables systems to respond dynamically to users’ emotional states by adapting content, providing empathic feedback, or delivering personalized therapeutic strategies with the goal of improving user engagement and treatment outcomes.

The third dimension concerns the system’s level of affective adaptivity. Passive systems simply detect or log emotional states without adapting their behavior; reactive systems adjust responses based on detected emotions, such as shifting tone; while active systems aim to guide the user in their emotional experience, by, for instance, offering personalized emotional coaching to promote regulation and behavior change. In the following, we will focus on the application of affective computing in interventions, as this often entails the technical implementations given in screening or diagnosis.

### 4.2 Patient-facing passive adaptivity

When considering passive patient-facing systems that aim to increase mental health by capturing emotional states, the primary application is digital self-monitoring. Research indicates that people who regularly monitor their emotions are better at managing stress and controlling emotional reactions (71, 72). Additionally, monitoring emotions over time can be part of screening or diagnosis as (early) signs of illnesses can be detected, which in turn allows for earlier and potentially more effective interventions. Conventional self-monitoring often requires individuals to manually track their emotions, behaviors, and surrounding circumstances using tools like paper diaries, mood logs, or structured symptom checklists, then, these records can later be examined with clinicians during therapy (73, 74). Despite their clinical value, such traditional approaches can be time-consuming, prone to memory inaccuracies, and often fail to capture emotional experiences as they happen.

Therefore, passive and semi-passive digital tools, including smartphones, wearable devices, and experience sampling apps are employed to track emotional states continuously and in real time (75). For example, studies using real-time emotional tracking methods have found that individuals with mood disorders experience greater emotional stability when they engage in self-monitoring (76) and adolescents that use mobile self-monitoring tools can reduce symptoms of depression (77).

**Figure 3:**
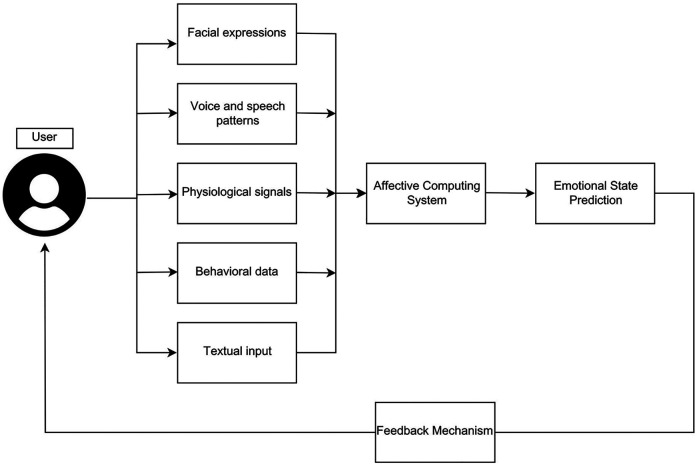
Affective computing systems in DMHI.

Affective computing systems, explained in Figure 3, leverage such multi-modal data sources. They integrate diverse streams of input such as facial expressions (e.g., detecting smiles, frowns, or expressions of surprise or distress), vocal tone (e.g., pauses, pitch, speed, and tone), physiological signals (e.g., heart rate variability, galvanic skin response, respiration rate, and EEG), and behavioral data (e.g., app usage, movement patterns via accelerometers, typing speed), and digital journaling inputs (textual capture of emotions, experiences, or personal thoughts using digital tools), to detect emotional states with greater accuracy and contextual awareness (1, 78). Affective computing technologies not only reduce the burden of manual tracking, but also facilitate self-awareness by enabling continuous data collection and trend analysis, and furthermore hold potential for early prediction of mental health deterioration or disease onset. The analysis results allow for instantaneous feedback, helping users to become aware of mood fluctuations and potential emotional triggers as they happen (79). Furthermore, users and clinicians can be notified of concerning trends, surpassing the delayed feedback typical of conventional approaches.

Therefore, machine learning algorithms are applied to identify mood patterns, behavioral signals, and emotional irregularities. For example, facial recognition algorithms can analyze micro-expressions to identify emotions like sadness or anger (80), while voice analysis tools detect affective cues from pitch, tempo, and intonation (81). Wearable devices can capture physiological signals such as heart rate variability or galvanic skin response, which are strongly correlated with emotional arousal and stress (82). Additionally, natural language processing can interpret emotional valence in textual or spoken language (83). Overall, a solid technological foundation exists, to detect affect from diverse modalities.

### 4.3 Patient-facing reactive adaptivity

While passive affective computing systems support emotional self-monitoring by detecting and presenting emotional states to users, reactive systems take this further by adapting their behavior in response to those detected emotions. This is achieved by analyzing affective data in real time and dynamically adjusting the system’s outputs, interface, or prompts based on the user affect. The adaptivity in speaking tone can be used to simulate empathy, as for instance, emotionally aware conversational agents like Wysa,^1^ and Replika^2^ to create safe environments that enable users to articulate their feelings and engage in structured emotional reflection (68).

One of the key approaches to improving emotional human-computer interaction is to simulate mirroring, which refers to the unconscious tendency to reflect or mimic another individual’s emotions. This psychological phenomenon can be leveraged in affective computing-based systems to assist users in self-reflection and emotional awareness. For example, Rajcic and McCormack (84) discuss how artificially intelligent systems equipped with mirroring capabilities can foster empathy and promote deeper emotional engagement.

In addition to mirroring, active listening constitutes another central strategy. Unlike passive reception, active listening involves attentively processing users’ verbal and emotional cues to facilitate meaningful exchanges of ideas and emotions. According to Roshanaei et al. (85), artificial intelligence (AI) that actively listens can better navigate empathy, making interactions more personalized and emotionally resonant. The importance of active listening in communication has also been emphasized by Bodie et al. (86), who highlights its central role in fostering understanding and rapport. Furthermore, Oertel et al. (87) propose an engagement-aware, attentive AI listener designed specifically for multi-user interactions, thereby extending active listening’s benefits to more complex social settings.

Technically, reactive systems often operate via rule-based logic or machine learning models that personalize feedback based on real-time affective data (88, 89). These adaptive mechanisms rely on predefined thresholds or models trained on longitudinal data to decide when and how to intervene. By aligning system behavior with users’ affective states, reactive systems help bridge the gap between emotional awareness and behavioral adjustment, providing more targeted and relevant support compared to passive feedback alone.

### 4.4 Patient-facing active adaptivity

In contrast to passive self-monitoring or reactive feedback systems, active affective computing systems go beyond recognizing and responding to emotional states: They intervene deliberately to guide users through structured emotional development processes. These systems are designed to promote behavioral change, emotional regulation, and therapeutic progress. On a short term basis, this can include recommending just-in-time micro-interventions, which are small behavioral suggestions that aim to increase the well-being in the moment. For instance, the system may recommend listening to AI-generated music based on the patient’s current emotion state (90), or a conversational agent can guide through mindfulness and breathing exercises (91) when stress is detected.

Long-term interventions are often drawing on established psychological frameworks like Cognitive Behavioral Therapy (CBT), where the users can receive personalized support based on AI-driven analysis of their emotional patterns, language use, and engagement levels (7), such as adaptive feedback, tailored coping strategies, and dynamically adjusted task difficulty (92). A prime example of active emotional adaptivity is the aforementioned Woebot, a CBT-based conversational agent^3^ that offers users structured mental health support by combining mood tracking with real-time psychoeducation and cognitive restructuring techniques. Unlike reactive systems that offer situational prompts, Woebot initiates therapeutic exercises, challenges cognitive distortions, and tracks progress over time, representing an active attempt to influence emotional and behavioral outcomes.

Another important emotional adaptive approach is emotion regulation coaching. Shi (93) present frameworks in which AI assists users in managing their emotions effectively through personalized coaching techniques. It has been also extensively reviewed by Sadka and Antle (94), who demonstrate its widespread application across different domains. Furthermore, empirical studies provide evidence of the effectiveness of AI-driven emotion regulation coaching applied in schools, where such systems help cultivate an adaptive emotional atmosphere conducive to learning and well-being (95, 96).

Additionally, Badia et al. (97) proposed the Emotional Labyrinth, an architecture designed for affective-driven procedural content generation in virtual reality environments, aimed at facilitating more effective emotional regulation. Alipour et al. (98) explored Model-Free Reinforcement Learning mechanisms through which these systems can induce behavioral change in users. For example, the adaptive user interface can intelligently guide users toward safe areas during emergency training by responding to their emotional states, helping them make calm and effective decisions under stress. Numerous efforts have focused on these aspects, with comprehensive surveys of user-centered design approaches and evaluation methodologies provided by Martins et al. (99). Owing to their adaptability, such systems are more readily accepted by users and yield considerable gains in both task performance and perceived usability.

Moreover, affective computing can be leveraged to directly target the treatment of emotional disorders (100), and patient surveys indicate a proportion of openness to such collaboration in mental health care (101, 102). Research includes methods based on multimodal data, gated sequential modeling architectures to extract continuous features of the data, and end-to-end learning systems with video as the direct input and emotion as output (19, 103). Such AI is now being incorporated into therapeutic interventions themselves and offering support (104). These systems can deliver CBT modules, track patient mood over time, and even provide real-time feedback during sessions, which in turn strengthens the foundation for personalized and adaptive therapy, as emotional insights enable therapeutic systems to dynamically tailor interventions to an individual’s current affective state, thereby enhancing treatment relevance and engagement (78). This shift also highlights the technological capabilities of AI in the broader landscape of mental health treatment, reshaping how care is accessed and delivered.

### 4.5 Therapist facing applications

While digital mental health interventions are generally conceptualized as direct-to-user technologies, therapists remain the conventional and central providers of psychological care. Therefore, a system is considered therapist-facing when the core interpretation and decision-making responsibilities lies with the therapist. Also, in these cases affective computing can be used for improvement.

Effective and timely intervention in mental health care relies heavily on continuous monitoring of patients, as explained in a previous section. This monitoring can be done in-session and out-of-session. Remote monitoring performed out-of-session allows the therapist to gain new insights beyond the boarder of their direct contact with the patients. Recent advancements have introduced wearable technologies integrated with AI, such as electronic skin devices, which can continuously monitor physiological indicators like cortisol levels and skin conductance to assess stress responses (8). The passive monitoring of emotional states during daily activities, such as studying or working, provides the clinician with a broader context for intervention (105). With in-session monitoring affective computing can enhance the therapeutic process by analyzing unstructured clinical session data using models such as ChatGPT (106) to provide the clinicians with real-time insights into patient emotions as the session progresses (107, 108).

Therapeutic support can also be provided remotely through teletherapy: The mental health services and counseling that are provided via the video or phone call. It enables the delivery of care beyond traditional in-person sessions. Teletherapy not only facilitates continuous monitoring of patients when they are not physically present but also offers real-time feedback and intervention during virtual interactions. Affective computing models have been increasingly integrated into teletherapy platforms to enhance these capabilities, enabling more scalable mental health care delivery by for instance providing real-time facial emotional recognition (109). Lenartowicz (110) reviewed the role of AI in teletherapy, emphasizing its potential to improve accessibility and convenience, including the use of virtual reality (VR) interventions to target specific psychological conditions. For instance, simulating feared environments to help treat phobias, or providing immersive scenarios to support PTSD therapy (111, 112). Therefore, research and implementation of AI-assisted teletherapy continue to progress (113).

Furthermore, previous studies have explored potential in assisting clinicians by recommending personalized therapy through large-scale data analysis (68, 114). Deep learning approaches have been investigated for selecting appropriate therapies such as CBT and predicting the relationship between CBT and social anxiety to inform future treatment choices (115). A notable example is that facial expression-based depression detection methods have been proposed to enhance depression diagnosis and improve the quality of counseling (116). Overall, these use-cases show that affective computing does not only carry a lot of potential in directly interacting with patients to improve their well-being, but also to support clinicians in their work.

### 4.6 Clinical evaluation

As affective computing-based DMHIs become more integrated into mental health applications, clinical evaluation of their appropriateness and effectiveness in outcomes is central. However, only a small number of studies have clinically assessed recent solutions. An overview of performed clinical evaluations is presented in Table 2. For example, Heinz et al. (3) conducted a randomized controlled trial using a chatbot named Therabot with individuals experiencing depression, anxiety, or eating disorders. Results indicated significant reductions in depressive and anxious symptoms, with improvements in emotional recovery and therapeutic alliance comparable to traditional psychological treatments. Similarly, (68) demonstrated that Woebot reduced symptoms for depression and anxiety significantly within two weeks. Likewise, the chatbot Wysa was found effective in real-world usage, lowering psychological distress through guided journaling and reflective dialogue (9). Limbic Access, deployed in the United Kingdom’s National Health Service, utilizes AI-driven assessments to support clinicians based on patient text inputs and clinical records to screen for depression, anxiety, and PTSD while offering a conversational agent to improve recovery rates (4). In the case of psychosis, Avatar Therapy (2) employs virtual avatars to represent hallucinatory voices; randomized controlled trials demonstrate that this approach can significantly reduce distress and hallucination frequency compared to standard treatment.

**Table 2:** Clinical evaluations of representative affective computing applications for mental health.

Application	Targeted condition	Used data	Intervention strategy	Evaluation type
Avatar therapy (2)	Psychosis (auditory hallucinations)	User-created avatars representing internal voices	Therapist-guided conversations with avatars to reduce distress	Clinical trials in the UK; participants reported improved mood and reduced voice severity after 16 weeks
Limbic access (4)	Depression, anxiety, PTSD	Text-based inputs during e-triage assessments	AI-driven diagnostic support for clinicians	Deployed in UK health systems; over 210,000 patients screened with 93% accuracy
Therabot (3)	Depression, anxiety, eating disorder	Chat inputs (text)	CBT chatbot responses	RCT
Woebot (68)	Depression, anxiety	Chat inputs (text)	CBT chatbot responses	RCT
Wysa (9)	Psychological distress	Textual journaling, app interactions	AI-led reflective dialog, coping suggestions	Real-world study

Applications for monitoring, which have been evaluated for performance rather than clinical outcome, include PhysioFormer, which integrates physiological signals (e.g., electrocardiogram and electrodermal activity) from the WESAD dataset to detect stress and affective states, achieving close to perfect classification accuracy in controlled lab settings (117) clearly indicating an overly optimistic experimental design, and FacePsy, a mobile tool, which analyzes facial expressions and eye-tracking data to identify depressive symptoms, shows promising clinical accuracy (AUROC 81%) in patient trials (118). MoodRhythm, which was assessed in a pilot study (119), combines passive sensing (e.g., GPS, accelerometer) with mood self-reports to provide rhythm-based mood feedback, leading to better self-awareness in bipolar patients. Overall, the actual application of affective computing-based systems in mental healthcare is only emerging. While the technological performance is increasing, large scale studies on the effect on clinical outcome and user engagement are missing.

### 4.7 Ethical concerns and considerations for clinical applications

The gap between affective computing research and its clinical application is besides ethical skepticism rooted in limited technological performance. Although, reviews for specific applications like monitoring mental health with smart wearable report high performance values ranging from 78%–97% (120), more recent work criticizes the lack of standardized approaches to validate systems, which complicates independent verification and comparison of systems, and also empathizes that current emotion recognition models often perform well under controlled laboratory conditions but face significant performance reduction when applied to in-the-wild data (121). Furthermore, Monteith et al. (122) caution that the commercial deployment of emotion AI may exacerbate social stigma and discrimination because AI models may be biased. Similarly, Hernandez et al. (123) point out that the increasing scale of AI deployment and the shift in agency from experts to lay users, coupled with regulatory shortcomings, may lead to unforeseen risks. A related concern is raised by Shimo (124), who argue that current affective AI systems are often developed and deployed under the assumption of minimal variation in emotional expression across human populations. This limited understanding of human and cultural diversity may compromise the systems’ ability to accurately recognize and interpret the emotions of marginalized or underrepresented groups. From a data ethics perspective, Durovic and Corno (125) emphasize the privacy risks posed by affective computing-based systems, particularly due to its reliance on large volumes of personal data for training and inference. In addition, Chu et al. (126) explore the psychological implications of emotional attachment to AI, warning that perceived relationships between users and machines could lead to confusion and emotional dependency. More critically, Devi et al. (127) suggest that such attachments may even pose threats to personal identity and self-conception.

Besides the technical limitations large ethical concerns exist, particularly regarding patient privacy, informed consent, and emotional manipulation (128, 129). Moreover, a critical debate persists regarding whether AI systems can genuinely exhibit authentic emotions. Vyas (130) has investigated the levels of trust and satisfaction among AI users, revealing complex and nuanced perspectives on this issue. Moreover, Glikson and Woolley (131) and Yang and Rau (132) provide comprehensive reviews of the literature on human trust in affective computing systems, demonstrating that the display of adaptive emotional responses by AI can considerably influence users’ trust, with appropriately aligned emotional expressions generally enhancing perceived trustworthiness and user satisfaction. However, discrepancies or perceived inauthenticity in the AI’s emotional adaptation may foster skepticism and ultimately diminish user trust in the system (131). Similarly, Rubin et al. (133) recently found that identical empathic responses are rated more empathic and supportive when believed to be human-generated than AI-generated, highlighting potential challenges in integrating affective computing systems into emotional caring situations.

Furthermore, the display of emotions does not only influence the user’s trust, but also their engagement. Yu et al. (134) examined the effects of emotional displays on user engagement, demonstrating that emotionally expressive content can enhance user attention and promote a wider range of products through visually captivating designs. Similarly, Maduku et al. (135) investigated the relationship between customer emotions and engagement in the context of AI assistant usage, concluding that positive emotional experiences and engagement with digital voice assistants considerably influence customer loyalty. In line with these findings, Chang and Herath (136) showed that AI systems capable of recognizing, interpreting, and responding to human emotions can effectively strengthen emotional engagement and foster greater trust in human-AI interactions. Beyond assessment of the users, the opinion of clinicians is of high importance, as they can recommend and apply systems. Research conducted in Japan indicates that attitudes toward the use of affective computing systems in healthcare positively correlate with individuals’ familiarity with the technology (137). On a global scale, Doraiswamy et al. (138) found opinions from 791 psychologists from 22 different countries showing that while healthcare professionals do not oppose the integration of such tools, many maintain that these technologies are unlikely to replace human clinicians in delivering truly empathetic care.

In order to improve the application of affective computing, Mohammad (139) provided a structured ethical checklist, highlighting the risks of privacy violations, emotional exploitation, and societal division. He advised integrating fairness, accountability, and explainability at early design stages. Saeidnia et al. (140) further argues that AI-driven emotional interventions should embed ongoing ethical review processes and stakeholder participation. They also call for regular assessments of algorithmic bias, data protection, and stratified impact. In response to these issues, regulatory bodies worldwide are gradually introducing relevant legislation (141). Furthermore, Löchner et al. (142) propose the TEQUILA framework to guide responsible development of digital mental health interventions, and Latif et al. (143) present promises and perils of AI-based emotion recognition to encourage prosocial development. Consequently, future efforts must prioritize improving emotion detection accuracy in-the-wild, while maintaining ethical standards and user privacy, as well as ensuring a level transparency that increases patient and clinician’s trust.

## 5 Future directions

With the ongoing evolution of affective computing in digital mental health, we suggest that future development should prioritize four key directions: multimodal interaction, explainable modeling, personalized models, and integration with healthcare systems.

First, **multimodal** input and output will be central to the next stage of development, as affective content is distributed across multiple channels. Compared to unimodal systems, multimodal architectures offer improved recognition accuracy and better contextual awareness (144). Nevertheless, this introduces the challenge of fusing incongruent modalities; for example, a patient may verbally express that they are feeling good but at the same time shake their head. That said, this obstacle can still carry potential, as from a psychological perspective such incoherencies can indicate discrepancies between expressed and experienced emotions, or conflicting self-states (145), and might therefore highlight content that is of therapeutic interest. On the other hand, multimodal output can introduce embodied agents, such as social robots or avatars that respond to the detected emotions through multiple channels like facial expressions, gestures, and gaze while answering verbally. Such agents may serve as empathic companions, co-therapists, or emotion regulation aids, supporting users during stress or therapy by mirroring affect or maintaining calming behavior. Research suggests they can improve engagement, reduce loneliness, and support emotional awareness, particularly in vulnerable populations such as children or older adults (93). However, such systems introduce new risks such as over-attachment and emotional dependency, which require careful application.

Second, **explainable AI** is essential in digital health applications. In clinical systems, lack of explainability can reduce clinicians’ trust in system outputs and hinder patients’ understanding and acceptance of recommendations, ultimately affecting treatment outcomes. Explainable models improve transparency, fairness, and communication efficiency, support shared decision-making, and promote patient engagement (146). Existing studies have explored various techniques such as visualizing attention in medical imaging in order to understand the central parts of the AI’s decision (147), as well as salient features and counterfactual explanations to prevent clinicians from over-relying on incorrect AI outputs (148). However, when applied to some mental illnesses (such as anxiety and depression) where the primary modalities are audio, physiological signals or brief facial micro-expressions, general-purpose explainable AI methods often struggle. These modalities lack visual structures, making outputs hard to interpret and inaccessible to non-technical users (149). There is thus a high need for task-, modality-, and user-specific explainability frameworks that produce clinically relevant explanations.

Third, **personalized modeling** is critical to long-term effectiveness and fairness in affective computing systems, as emotional responses vary considerably across individuals, influenced by factors such as demographic attributes and cultural contexts (150). Likewise, mental disorders like depression can manifest differently across cultures (151). Additionally, mental illnesses like schizophrenia can affect the display of emotions, where in the case of flat affect, there is barely or no external emotional display, while the subjective experience of emotions is not diminished (152). Such individual and disease-specific differences need to be accounted for, as uncertainty in emotion recognition could lead to inappropriate emotional adaptation from the system. Additionally, mental disorders have an individual progression, which is reflected by the moving target problem (153). The user’s emotional and mental state evolves due to therapeutic progress, life events, or symptom fluctuation, as is the case for flat affect in patients who have suffered from psychosis (154). Although these adaptations to culture, disease, and personal progression are central for a high-quality therapy, such specifications of affective computing models are still only under development and require further research.

Finally, the **clinical deployment** of affective computing systems is lacking behind the start-of-the-art in research and requires deeper exploration at both system integration and application levels. Future models should interface with electronic health records, support telemedicine platforms, and be embedded within intervention planning workflows (155). Ensuring closed-loop mechanisms for data standardization, privacy compliance, and clinical acceptability will be essential to integration. Recently, privacy-preserving federated learning frameworks have been introduced into physiological and speech-based emotion recognition. These architectures allow model training across distributed devices without sharing raw data, enabling a balance between data protection and cross-device generalization (156), and therefore pose a promising approach for DMHIs. Furthermore, an increase in clinical deployment requires clinical validation and large-scale longitudinal studies in order to ensure therapeutic value. Concluding, the application of affective computing for mental health requires further technical improvements such as multimodal integration and personalization, as well as an increase in explainability and safety to increase trust, while clinical trials are outstanding to ensure an actual benefit of the application of affective computing.

## 6 Discussion and conclusion

This review has outlined how affective computing technologies are increasingly integrated in digital mental health interventions, spanning both patient- and therapist-facing applications. These systems aim to recognize, interpret, and respond to users’ emotional states in real-time to support self-monitoring, enhancing engagement, and enable more personalized and adaptive interventions (107–109). A central distinction in this review is between passive, reactive, and active adaptivity. Nevertheless, the boundaries between these types are often blurred in practice. Despite increasing sophistication, emotion detection technologies still face validity and reliability concerns, especially across diverse populations. Current algorithms often struggle with cultural variation, individual differences, and context sensitivity (121, 124). Without accurate and interpretable emotion recognition, downstream interventions may misfire and therefore undermine user trust and therapeutic efficacy (131, 132).

While much attention in digital mental health has focused on direct-to-user tools, we highlight the growing role of therapist-facing applications. Affective computing technologies can support clinicians by offering real-time emotional insights, informing diagnosis, enabling personalized care, and extending monitoring beyond therapy sessions (4, 107). Such hybrid models preserve the therapist’s expertise while enriching it with algorithmic support. However, these systems raise new questions around accountability, interpretability, and the therapist’s role in AI-augmented decision-making (110, 139). If affective feedback is incorrect or biased, how should clinicians interpret or override it? Integration of explainable AI methods and clinician-in-the-loop frameworks will be essential for ensuring these systems remain clinically meaningful and ethically deployable (140).

Moreover, large ethical challenges are being faced, as empathetic AI responses may risk emotional dependency or manipulation, especially when systems are designed to mimic human emotion (126, 127). Moreover, the commercial deployment of affective systems raises concerns around bias, privacy, and social inequality, since models trained on non-diverse data may misinterpret emotions in marginalized groups (122, 124), while real-time affective monitoring could impose surveillance risks if not properly regulated (125). Additionally, commercial mental health applications face the risk of being designed in a way that keeps users attached in order to increase customer numbers instead of designing systems that aim to promote recovery in the fastest and safest way. Consequently, transparency in AI systems as well as their development should be of highest interest, to ensure safe deployment with high clinical value.

In the future affective computing will likely play an increasing role in both patient-facing interventions and clinical workflows. Key directions for development include multimodal architectures, explainable and personalized models, and integration with healthcare infrastructure. In particular, embodied agents, such as emotionally expressive avatars and robots, may offer new modes of support by combining affective sensing with naturalistic, human-like interactions (93). Nevertheless, affective computing technologies should be viewed not as replacements for human care, but as tools to enhance and extend it. To enhance the trust, clinical evaluation of the effect of affective computing on the clinical outcome require extensive attention in the future, as it has only been done scarcely so far. Concluding, the success of affective computing for mental health will depend on interdisciplinary collaboration across AI, psychology, and clinical practice, as well as robust evaluation frameworks.

## Footnotes

^1^ https://www.wysa.com/

^2^ https://replika.com/

^3^ https://woebothealth.com/
